# Bandgap modulation in the two-dimensional core-shell-structured monolayers of WS_2_

**DOI:** 10.1016/j.isci.2021.103563

**Published:** 2021-12-03

**Authors:** Seohui Kang, Yonas Assefa Eshete, Sujin Lee, Dongyeun Won, Saemi Im, Sangheon Lee, Suyeon Cho, Heejun Yang

**Affiliations:** 1 Department of Chemical Engineering and Materials Science, Graduate Program in System Health Science and Engineering, Ewha Womans University, Seoul 03760, Republic of Korea; 2 Department of Energy Science, Sungkyunkwan University, Suwon 16419, Republic of Korea; 3 Department of Physics, Korea Advanced Institute of Science and Technology (KAIST), Daejeon 34141, Republic of Korea

**Keywords:** Materials science, Materials synthesis, Nanomaterials

## Abstract

Tungsten disulfide (WS_2_) has tunable bandgaps, which are required for diverse optoelectronic device applications. Here, we report the bandgap modulation in WS_2_ monolayers with two-dimensional core-shell structures formed by unique growth mode in chemical vapor deposition (CVD). The core-shell structures in our CVD-grown WS_2_ monolayers exhibit contrasts in optical images, Raman, and photoluminescence spectroscopy. The strain and doping effects in the WS_2_, introduced by two different growth processes, generate PL peaks at 1.83 eV (at the core domain) and 1.98 eV (at the shell domain), which is distinct from conventional WS_2_ with a primary PL peak at 2.02 eV. Our density functional theory (DFT) calculations explain the modulation of the optical bandgap in our core-shell-structured WS_2_ monolayers by the strain, accompanying a direct-to-indirect bandgap transition. Thus, the core-shell-structured WS_2_ monolayers provide a practical method to fabricate lateral heterostructures with different optical bandgaps, which are required for optoelectronic applications.

## Highlights

• Core-shell-structured WS_2_ monolayers with modulated bandgap are grown by CVD
• Doping and strain effect in band structure are revealed by Raman and PL spectroscopy
• Through DFT calculation, we showed that the bandgap can be tuned by the strain
• Core-shell-structured WS_2_ with different bandgap provides option to tune the bandgap

## Graphical abstract

**Figure:**
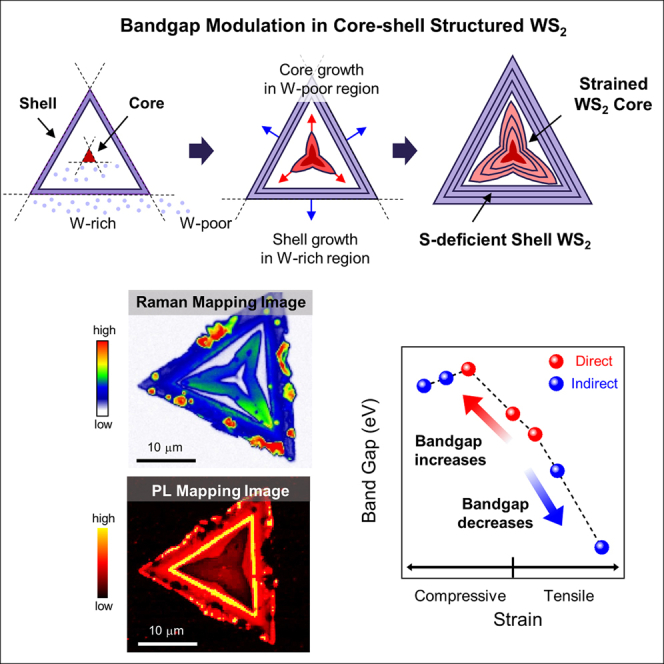

## Introduction

Two-dimensional (2D) atomic crystals, such as graphene, hexagonal boron nitride (*h*-BN), and transition metal dichalcogenides (TMDs), have recently received considerable attention because of their unique 2D characteristics in physics and chemistry, which are promising for future optoelectronic devices (Radisavljevic et al., 2011; Wang et al., 2012; Lopez-Sanchez et al., 2013; Jariwala et al., 2014). Among the various 2D atomic crystals, group 6 TMDs are considered as attractive semiconducting materials owing to their tunable bandgaps and layer-dependent exciton dynamics (Chhowalla et al., 2013; Ding et al., 2011). Hexagonal tungsten disulfide (2H-WS_2_) is a semiconductor in group 6 TMDs, where its layers are weakly bonded by van der Waals interactions (Chhowalla et al., 2013; Butler et al., 2013). The 2H-WS_2_ exhibits an indirect bandgap in its bulk and undergoes an indirect-to-direct bandgap transition as it is thinned down to a monolayer (Gutiérrez et al., 2013; Yun et al., 2012). The electronic band structures of WS_2_ critically depend on the condition of the sample synthesis; thus, rigorous studies of the WS_2_ grown by various processes are required.

Confocal PL and Raman spectroscopy are suitable techniques for characterizing 2D layered materials. Monolayer WS_2_ has strong and distinct PL and Raman spectra owing to its direct bandgap. The uniform PL signal in a WS_2_ flake indicates its highly crystalline structure (Gutiérrez et al., 2013; Cong et al., 2014; Peimyoo et al., 2013), which is preferred for transistor applications. Nevertheless, WS_2_ monolayers grown by CVD exhibit various PL signals determined by the synthetic conditions; for example, it was reported that PL spectra were abnormally enhanced by abundant adsorbents that were formed during CVD growth (Peimyoo et al., 2013; Guiterrez et al., 2013; Liu et al., 2016a, ; Jeong et al., 2017; Sheng et al., 2017; Hu et al., 2019).

The role of strain to modify electronic structures of 2D TMDs have been intensively studied by confocal PL and Raman spectroscopy (Conley et al., 2013; He et al., 2013; Zhu et al., 2013). When a tensile or compressive strain is applied on TMDs by using a stretchable or bendable substrate, the bandgap of the TMDs significantly changes and direct-to-indirect bandgap transition has been reported (Wang et al., 2015; He et al., 2016). In previous studies on CVD-grown WS_2_, distinct intrinsic strains were introduced under different growth conditions; this was because of the different thermal expansion coefficients between the WS_2_ and substrates (Feng et al., 2017; Shi et al., 2019). The theoretical calculations have also predicted that strain modifies the valence band maximum (VBM) and conduction band minimum (CBM) of semiconducting TMDs such as 2H-WS_2_, which results in direct-indirect band gap transition (Yun et al., 2012; Wang et al., 2015; Shi et al., 2013; Chang et al., 2013; Desai et al., 2014; Johari and Shenoy, 2012; Peelaers and Van de Walle, 2012; Lu et al., 2012; Zhang et al., 2013; Maniadaki et al., 2016; Muoi et al., 2019). Accordingly, the growth mechanism in CVD remains to be further explored to understand and modulate the strain effects on the electronic properties of few-layered WS_2_.

Recently, heterogeneous 2D TMDs grown by CVD have been reported with various geometries (Jeong et al., 2017; Chen et al., 2014; Wang et al., 2018; Shinde et al., 2018). Jeong et al. reported WS_2_ with a hexagonal geometry that is segmented into alternating triangle domains: S-deficient and W-deficient domains (Jeong et al., 2017). The heterostructures with core metal oxides and shell TMDs have been found in a fullerene-like shape (Cain et al., 2016). Such TMDs show the multiple domains of the core and shell where nucleation- and diffusion-dominated growth processes coexist (Withanage et al., 2020). The core-shell structure growth by CVD offers a unique technique for engineering electronic heterostructures for diverse optoelectronic applications with TMDs (Fang et al., 2020; Jo et al., 2019).

In this study, we conducted thorough PL and Raman spectroscopy on core-shell-structured WS_2_ monolayers grown by CVD. The core-shell-structured WS_2_ monolayer possessed multiple domains that were optically distinguished as a core and surrounding-shell domain. Our WS_2_ monolayers with core-shell structures showed the prominent Raman modes of conventional WS_2_, 2LA, E′_2g_, and A_1g_. However, in contrast to conventional WS_2_ monolayers with PL peaks at 2.02 eV, the core-shell-structured WS_2_ exhibited weakened PL peaks located at 1.83 eV and 1.98 eV in the core and shell WS_2_, respectively. DFT calculations demonstrated that the lateral strain in the WS_2_ monolayer, originating from its unique 2D geometrical growth, generated the practical modulation of the optical bandgap observed in the core and shell domains. Our study suggests that the subtle modulation of the optical bandgap can be realized by introducing strains in the CVD process, which provides unique lateral heterostructures for future optoelectronic devices. Lateral heterostructures with different bandgaps will realize a simple design of multi-functional optoelectronic devices, which is distinguished from previous studies on WO_3_/WS_2_ devices. For that purpose, our WS_2_ monolayers grown by the 2D core-shell growth mode generate proper vacancies and strains in the 2D lattice, which is a novel engineering approach for future device applications, such as LED, photodetectors, and bipolar junction transistors (Kang et al., 2013; Pospischil et al., 2014; Baugher et al., 2014; Ross et al., 2014; Gong et al., 2013; Kim et al., 2021).

## Results and discussion

**Figure 1:**
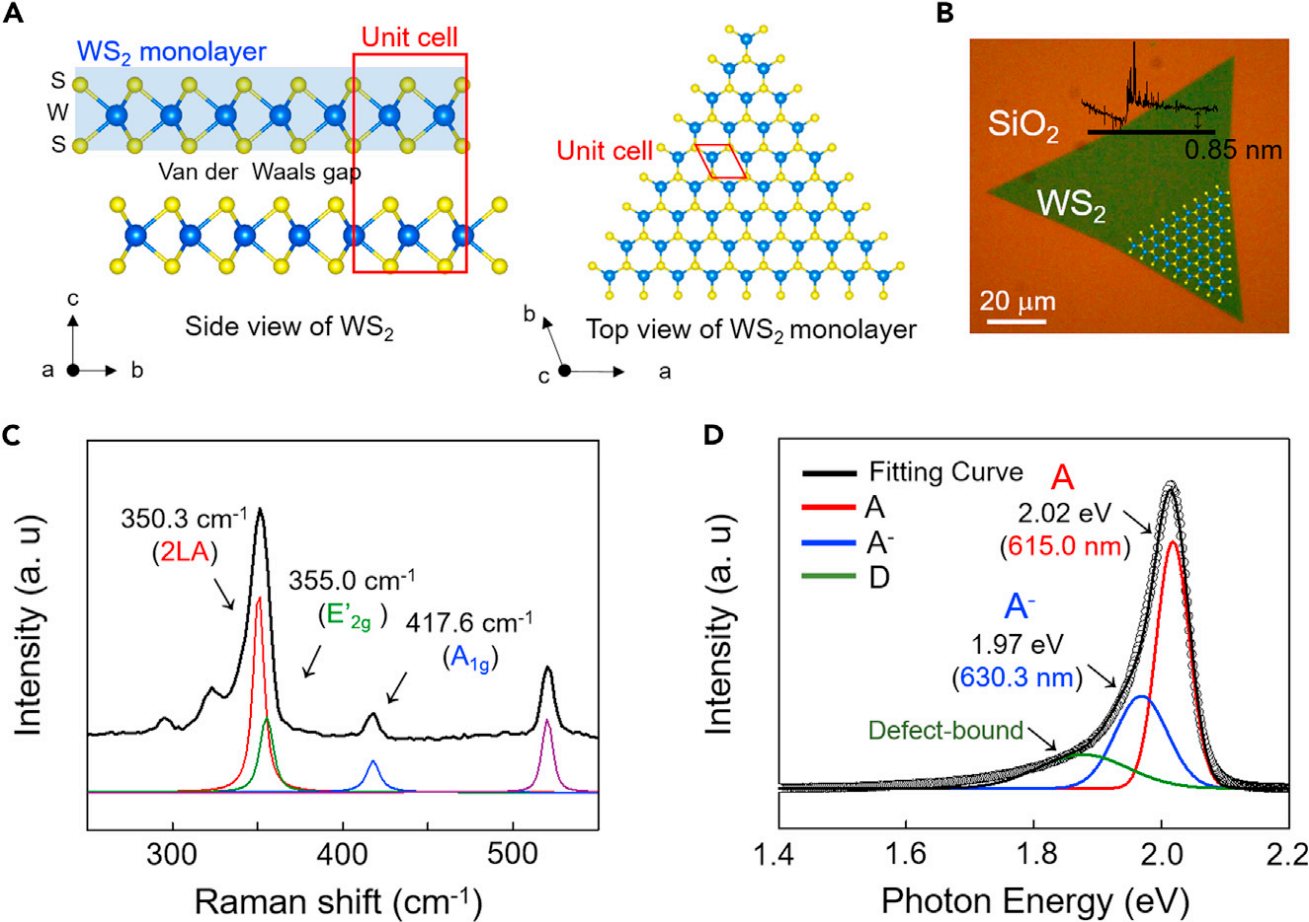
Single-domain WS_2_ grown by chemical vapor deposition (A) Crystal structure of hexagonal WS_2_. Side view of the WS_2_ and top view of the WS_2_ monolayer with a unit cell (red rectangles). (B) Optical image of the WS_2_ monolayer with a profile of thickness, which is estimated as 0.85 nm by AFM measurement. (C and D) (C) Raman and (D) PL spectra of single-domain WS_2_ with fitted curves. In the PL spectra, the red, blue, and green fitting curves labeled with A, A^−^, and D represent emission of exciton, trion, and defect-bound exciton, respectively.

As shown in Figure 1A, a three-atom-thick WS_2_ monolayer consists of a W atomic layer sandwiched between top and bottom S atomic layers. Semiconducting WS_2_ is referred to as 2H-WS_2_ because two WS_2_ atomic layers form a hexagonal unit cell. As shown in the top view of the WS_2_ monolayer, the W and S atoms were arranged by a triangular lattice structure, showing the hexagonal symmetry of WS_2_. The as-grown WS_2_ monolayer possessed a triangle shape with a thickness of 0.85 nm, as shown in the optical image and AFM height profile in Figure 1B. Raman and PL spectroscopy confirmed that the WS_2_ monolayer possessed a single domain owing to its uniform signals. As shown in Figure 1C, the single-domain WS_2_ monolayer shows three main Raman peaks at 350.3 cm^−1^, 355.0 cm^−1^, and 417.6 cm^−1^, which is consistent with the reported Raman active modes of WS_2_; the three peaks correspond to the 2LA (second-order longitudinal acoustic Raman mode at point M), E′_2g_, and A_1g_ of WS_2_, respectively (Berkdemir et al., 2013).

The PL spectrum of single-domain WS_2_ monolayer was fitted by three Gaussian curves: exciton emission (red curves, marked by “A”), negative trion emission (blue curves, marked by “A^−^"), and defect-bound exciton emission (green curves, marked by “D”). The PL spectrum showed a strong exciton emission at 615.0 nm with a low full-width half-maximum (FWHM) of 19.1 cm^−1^, which corresponded to the previously reported exciton emission at 2.01–2.02 eV (Plechinger et al., 2015; Bellus et al., 2015; Zhu et al., 2015; Peimyoo et al., 2014). The small and broadened PL peak at approximately 630.3 nm with a high FWHM of 50.6 cm^−1^ can be attributed to the electron-doped negative trion peak of WS_2_ (Zhu et al., 2015; Peimyoo et al., 2014). The detailed Gaussian fittings for the Raman and PL spectra are summarized in the supporting materials (see Tables S1 and S2).

**Figure 2:**
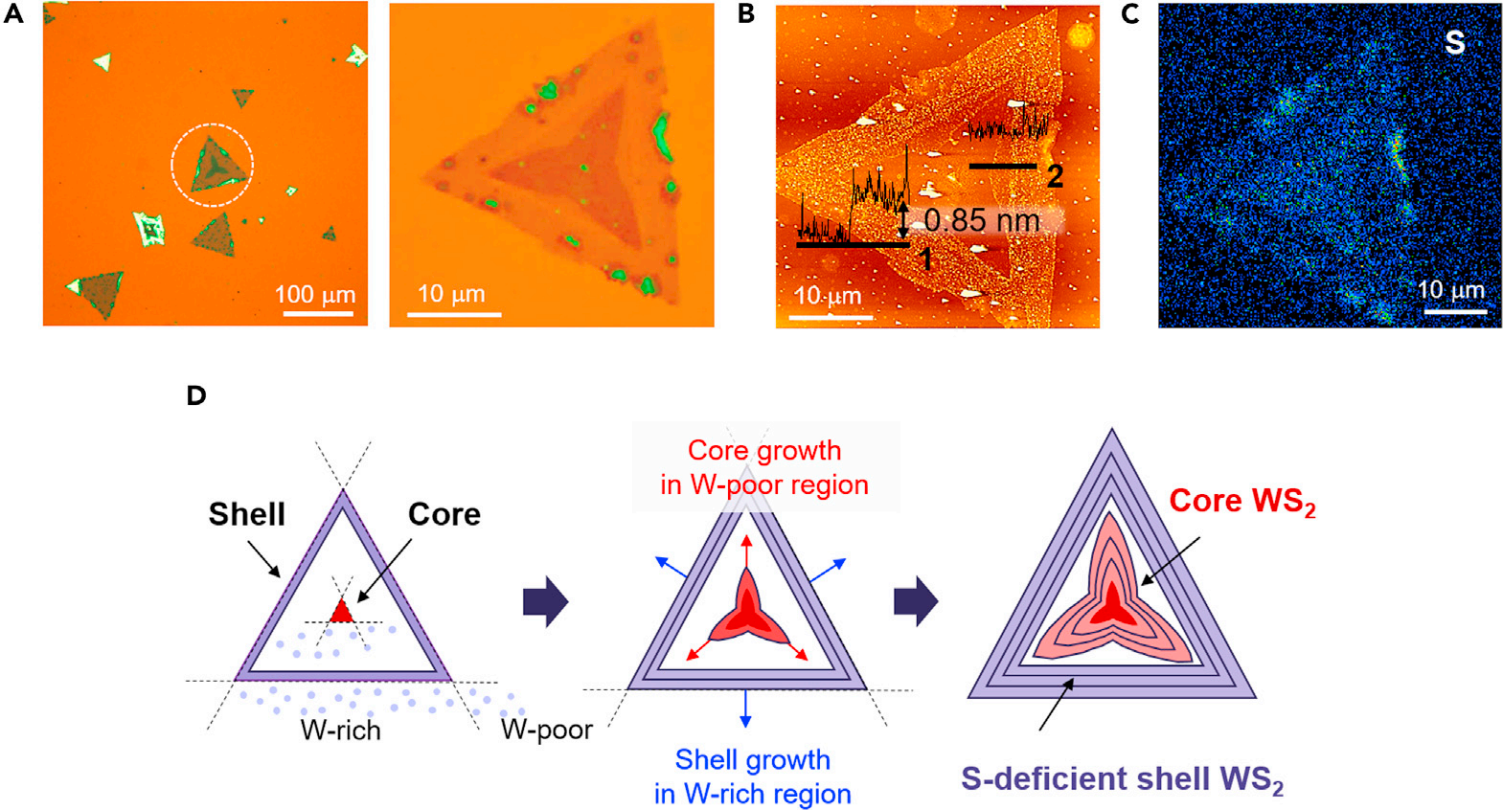
WS_2_ with multi-domains grown by core-shell growth mode (A) Optical images of the WS_2_ flakes with a triangular shape on a SiO_2_/Si substrate. A multi-domain WS_2_ flake is marked with a dashed white circle (left panel), and its magnified image is shown in the right panel. (B) AFM image of the multi-domain WS_2_ monolayer flake. Height profiles across the border of WS_2_ (line 1) and the different optical contrast region inside the WS_2_ flake (line 2) are shown in the AFM image. (C) EPMA mapping image of S in the core-shell WS_2_ monolayer. (D) Schematic images for core-shell growth mechanism for multi-domains WS_2_.

Although most CVD-grown WS_2_ samples have a single domain with a uniform thickness and optical contrast, as shown in Figure 1, multi-domain WS_2_ flakes are also found with a non-uniform optical contrast, as shown in Figure 2A. Both single- and multi-domain WS_2_ flakes were simultaneously grown on a substrate (see Figure S1 for more information). Although we continuously supply S source during the CVD growth, local variation of W and S sources leads to the formation of single- and multi-domain WS_2_ flakes. Optical microscopy showed that the interior core domain possessed a three-leg-starfish shape, and the surrounding shell domain possessed a triangular shape. Using AFM, we examined the thickness of the core-shell-structured WS_2_ and its change across the border between the core and shell domains. The WS_2_ flake with multiple domains was a monolayer with a thickness of 0.85 nm, as shown in the height profiles (line 1 in Figure 2B), without any change in the thickness across the border (line 2 in Figure 2B). The concentrations of W and S elements in the multi-domain WS_2_ were measured by using electron probe microscope analysis (EPMA). Although the elemental analysis was challenging with the sub-nanometer thickness of multi-domain WS_2_ monolayer (see Figure S2 in the supporting materials), we could observe the relative distribution of S as shown in Figure 2C; the core region has a little bit higher S concentration than the shell region, whereas the concentration of S is uniform within the domain except multilayer regions that are located at the shell boundary. The EPMA results demonstrate that the shell region has more S defects than the core region in our multi-domain WS_2_ monolayers.

It has been reported that core-shell-structured MoS_2_ monolayers can be synthesized by CVD with a similar geometry, as shown in Figure 2D (Zhang et al., 2017). According to the study, the growth of the core part of the WS_2_ is stimulated at the corner of its nucleus (depicted as a red triangle in Figure 2D), whereas the growth of the shell part begins from W-rich edges (depicted as a violet triangle shell in Figure 2D). The two different growth modes for the core and shell domains proceeded separately in W-poor and W-rich regions, generating core and shell structures with boundaries, as shown in the schematic of Figure 2D. We found that the ratio of W to S in the shell domain is relatively higher that the core domain, which indicates that our multi-domain WS_2_ monolayers have S-deficient shell domains. The core and shell domains are independently grown in our CVD growth via the 2D core-shell growth mode.

**Figure 3:**
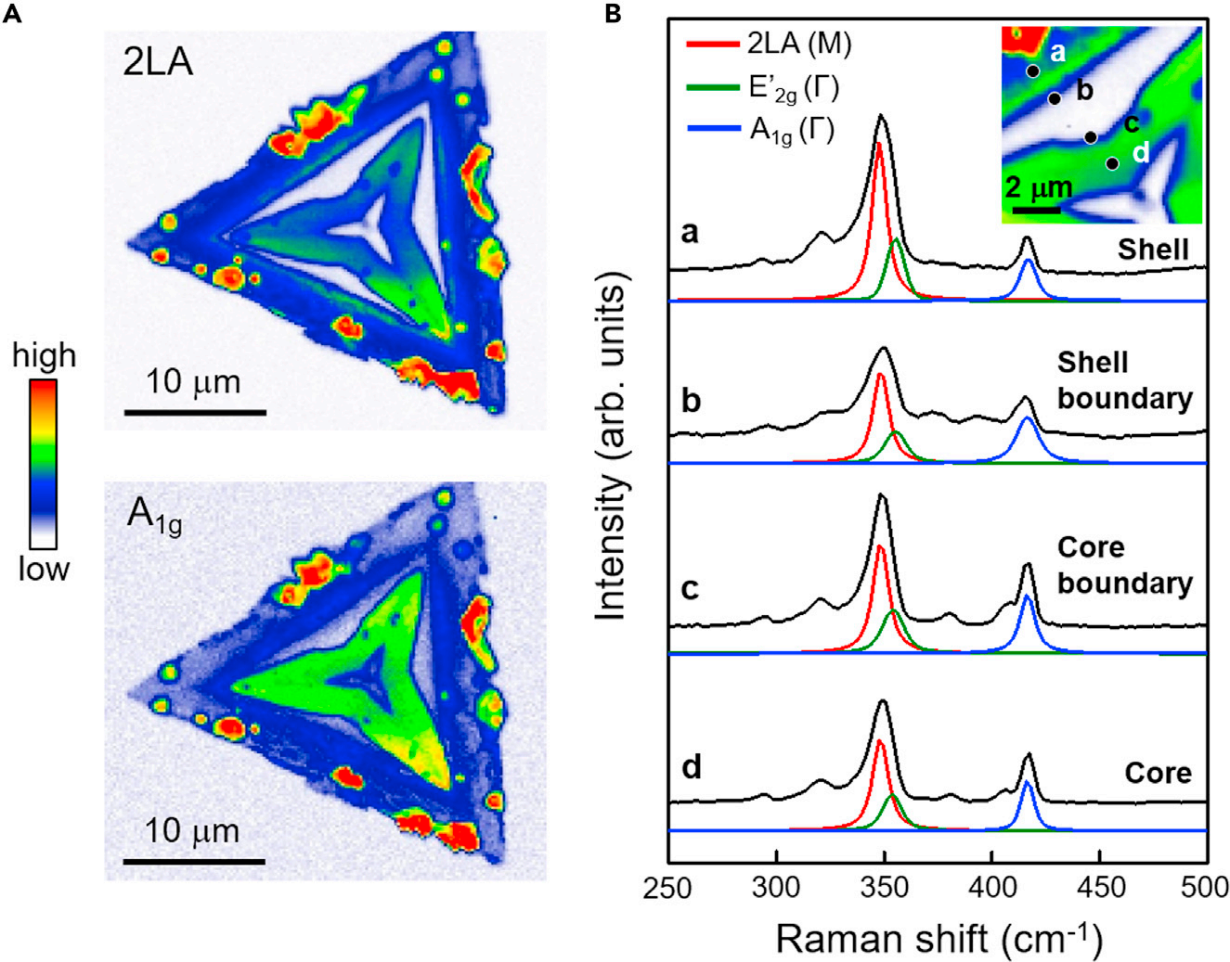
Raman mapping and spectra of core-shell-structured WS_2_ (A) Raman intensity mapping with two Raman active modes, 2LA (350.3 cm^−1^) and A_1g_ (417.6 cm^−1^), of WS_2_. (B) Raman spectra with fitted curves at the locations of *a*, *b*, *c*, and *d* in the inset of the Raman intensity mapping image. The Raman were fitted by multiple Gaussian curves, and the detailed fitting parameters are described in Table S1 in supporting materials.

The core-shell-structured WS_2_ monolayer was characterized by confocal Raman mapping with a step size of 250 nm. The entire area of the core-shell-structured WS_2_ monolayer showed the consistent Raman active modes of WS_2_, corresponding to the 2LA, E′_2g_, and A_1g_ modes of WS_2_. Figure 3A shows two Raman mapping images of a core-shell-structured WS_2_ monolayer (marked with a white circle in Figure 2A) with the peaks of 2LA and A_1g_. Based on the optical microscopy and Raman spectroscopy results, in this study, we divided the core-shell-structured WS_2_ monolayer into four regions: (1) shell, (2) shell boundary, (3) core boundary, and (4) core. The four regions are marked by letters from “*a*” to “*d*” in the inset of Figure 3B. We selected four points to represent each region whose Raman intensity and shift mapping are shown in Figure 3B and Figure S3, respectively. The Raman spectra from the four regions were analyzed by fitting them with Gaussian curves; the resulting fitting parameters are summarized in Table S1.

The region marked by “*a*” in the inset of Figure 3B, a WS_2_ shell domain, has Raman peaks at 347.6 cm^−1^, 355.0 cm^−1^, and 416.6 cm^−1^, which can be assigned to the Raman active modes of 2LA, E′_2g_ and A_1g_ in the conventional WS_2_. Compared with the WS_2_ monolayer with a single domain, the shell domain of the core-shell-structured WS_2_ monolayer exhibited a red-shifted A_1g_ mode by 1.0 cm^−1^ without any change in the E′_2g_ mode. Considering that the E′_2g_ and A_1g_ modes are sensitive to the strain and doping effect (Peimyoo et al., 2014; Wang et al., 2015; Conley et al., 2013; Zhu et al., 2013; Chakraborty et al., 2012; Nan et al., 2014), respectively, the red-shifted A_1g_ peak in the shell domain indicated effective n-type doping, probably due to sulfur deficiency. However, no significant strain effect was observed in the sample.

In the core domain of WS_2_, marked with the position *d* in the inset of Figure 3B, the Raman peaks exhibit red-shifted E′_2g_ and A_1g_ peaks by 1.3 cm^−1^ and 1.2 cm^−1^, respectively. In contrast to the unchanged E′_2g_ peak in the shell domain, the core domain exhibited a red-shifted E′_2g_ peak, which can be ascribed to the strain effect, particularly by tensile strain (Peimyoo et al., 2014; Wang et al., 2015; Conley et al., 2013; Zhu et al., 2013). The doping effect in the core domain was comparable with that in the shell domain because of the similar red-shifted A_1g_ Raman peak in the core and shell domains. The Raman features of our core-shell-structured WS_2_ monolayer are different from those of multi-domain hexagonal WS_2_ with a large blue-shifted A_1g_, which was explained by the polarity (p)-doping effect in the W-deficient domain (Jeong et al., 2017). Therefore, we interpret our results using the different p-doping and strain effects; the S-deficient shell domain possessed an n-type doping effect, whereas the core domain experienced a tensile strain together with an n-type doping effect.

Considering that defects could decompose TMDs (Fang et al., 2019; Gao et al., 2016), we confirmed the stability of our multi-domain WS_2_ monolayer. The confocal Raman and PL and optical microscope images of a same multi-domain WS_2_ flake in Figure S4 show that the sample is stable over two years without significant decomposition. It has been reported that most chalcogen vacancies (S deficiencies or defects) are passivated by oxygen atoms (Bui et al., 2015; Liu et al., 2016b). For example, Cui et al. recently reported that strong W-O bonds are responsible for the superior environment stability of WS_2_ by incorporating O atoms at the S vacancy sites of WS_2_ (Cui et al., 2021).

**Figure 4:**
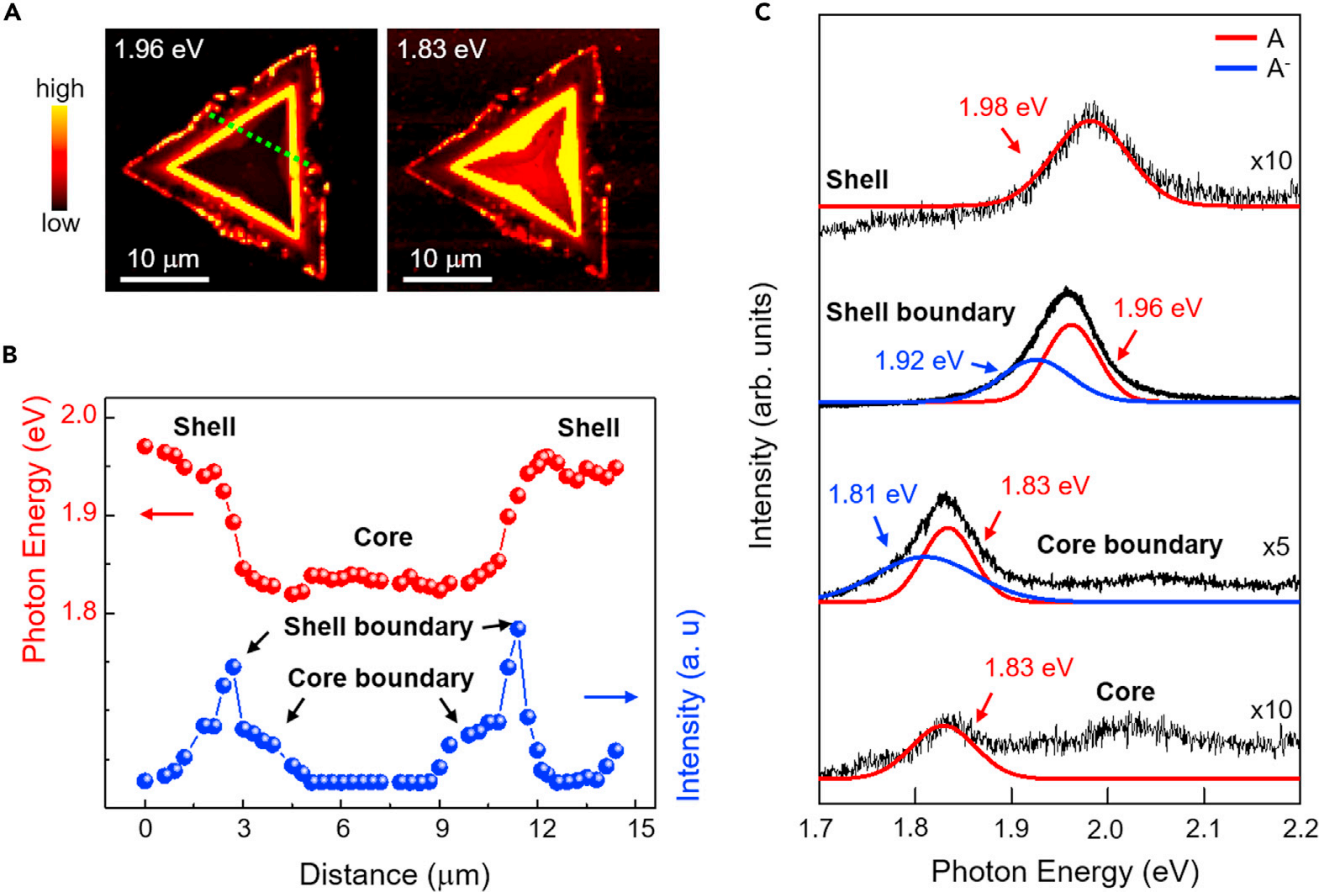
Photoluminescence study of core-shell-structured WS_2_ (A) PL intensity mapping with two photon energies, 1.96 eV and 1.83 eV. (B) Line profiles of the PL mapping image with exciton photon energy and PL intensity across the sample, collected along the dashed green line of Figure 4A. (C) PL spectra with fitted curves at the core and shell with PL spectra at the boundaries of the core and shell. The PL spectra were fitted by multiple Gaussian curves, and the detailed fitting parameters are described in Table S2 in the supporting materials.

Confocal PL spectroscopy revealed that the PL spectra of the core-shell-structured WS_2_ monolayer significantly varied at different locations in the flake, which is in contrast to a single-domain WS_2_ monolayer. As shown in Figure 4A, PL intensity mapping images show a large variation of PL signals between two exciton energies of 1.98 eV (at the shell region) and 1.83 eV (at the core region). Notably, the core and shell domains of the WS_2_ monolayer exhibited 5–10 times weaker PL emission than the boundary regions (see Figure S5). The line profiles of the PL mapping image shown in Figure 4B demonstrate that the PL intensity and exciton photon energy significantly change across the line profile in the core-shell-structured WS_2_ monolayer. The quenched PL spectra in the S-deficient shell domain can be explained by nonradiative carrier decay in defect-related states, which have been reported as deep trap sites in defective TMDs (Jeong et al., 2017; Liu et al., 2016a; Su et al., 2016). For the strain effect in the core domain of our core-shell-structured WS_2_ monolayer, the dramatically quenched PL spectrum centered at 1.83 eV can be explained by direct-to-indirect bandgap transitions, as often observed in strained TMDs (Wang et al., 2015; Conley et al., 2013; Lu et al., 2012; Kumar and Ahluwalia, 2013).

The boundaries between the core and shell regions in our WS_2_ flakes produced stronger PL spectra than the core and shell regions. The PL spectra were deconvoluted into two emissions, as shown in Figure 4C. The enhanced PL intensities at the boundaries can be explained by the chemical doping, oxidation, and chemisorption that occurred at the boundary region. Notably, several TMDs exhibited increased PL spectra with similar origins (Gutiérrez et al., 2013; Nan et al., 2014; Tongay et al., 2013; Sheng et al., 2017). The PL signals of the shell and core boundary comprised two sets of peaks (A and A^−^ peaks), as shown in Figure 4C. The energy difference of 0.043 eV for excitons and trions was reported in previous studies (Plechinger et al., 2015); thus, the two peaks (A and A^−^ peaks) in our PL spectra are attributed to the generation of excitons and trions in WS_2_.

Zhang et al. reported core-shell-structured MoS_2_ monolayers synthesized by CVD using a solid precursor of metal oxide (Zhang et al., 2017). In this study, the metal oxide precursor and S source provided a sufficient quantity of Mo and S in the growth process. Therefore, the core and shell domains were grown with a uniform chemical composition and merged into a flake with a boundary that was optically hidden but distinguished by PL spectroscopy. The enhanced PL intensity resulted from the p-doping and strain effects at the (optically) hidden boundary. Our core-shell-structured WS_2_ was synthesized under different conditions using a precoated hydrate W source on the substrate. Thus, a limited amount of W was supplied, and the WS_2_ monolayers could not merge their domains and boundaries having different chemical compositions.

**Figure 5:**
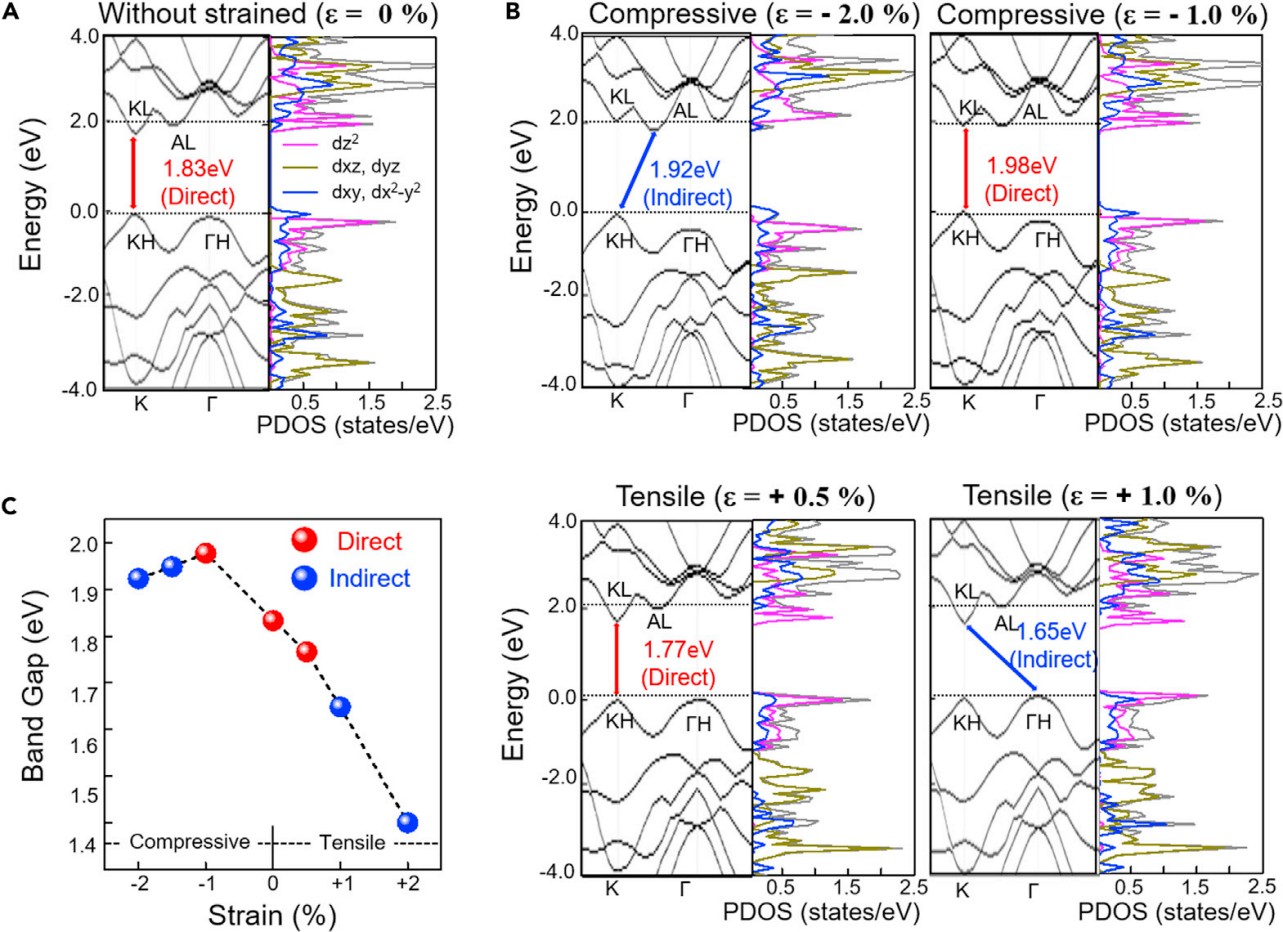
The calculated band structure and corresponding PDOS of monolayer WS_2_ under strain (A and B) (A) ε = 0% (unstrained), (B) ε = −1.0%, −2.0%, +0.5%, and +1.0%. (C) Calculated band gap of the strained WS_2_ monolayer as a function of strain.

Compression or tensile strains were found in many TMDs, mostly due to the formation of defects and dislocations during the CVD growth of the 2D geometry (Feng et al., 2017; Shi et al., 2019; Liu et al., 2014; Kataria et al., 2017). Previous studies have shown that the strains undergo direct-to-indirect bandgap transitions and changes in the emitted photon energy in the PL spectra of the TMDs (Yun et al., 2012; Wang et al., 2015; Shi et al., 2013; He et al., 2016; Chang et al., 2013; Desai et al., 2014). We conducted DFT calculations to show that the electronic structure of the WS_2_ monolayer could be modulated by the lateral strains. As shown in Figure 5A, a pristine WS_2_ monolayer without strain (ε = 0%) has a valence band maximum (VBM) and conduction band minimum (CBM) at the K point, indicating a direct bandgap semiconductor with a calculated bandgap of 1.83 eV. When a lateral strain (ε) was applied to the WS_2_ monolayer by changing the lattice constant *a* to ε*a* in the range from 0% to ±2.0% (positive ε for a tensile strain and negative ε for a compressive strain), the electronic structure of the WS_2_ changed. The VBM and CBM occurred at different symmetry points: KL, AL, and ΓH, depending on the strength of the strain (Figure 5B).

The bandgaps of the WS_2_ monolayer in the presence of strain, as estimated by DFT calculations, are summarized in Figure 5C. The bandgap of the WS_2_ monolayer increased in the range of −2% ≤ ε ≤ −1%, and thereafter, it decreased in the range of +0.5% ≤ ε ≤ +2%. Thus, the largest direct bandgap (1.98 eV) was obtained with a strain of −1.0%, where the bandgap of the WS_2_ monolayer decreased with strain accompanying the direct-to-indirect bandgap transition. Our DFT calculations exhibit strain-induced bandgap changes that are consistent with previous works. For example, it has been reported that the bandgap of WS_2_ monolayer decreases by a tensile strain, and strain-induced direct-to-indirect bandgap transition occurs as the tensile strain reaches 2.6% (Wang et al., 2015; He et al., 2016). In our DFT calculations, the decrease of bandgap and its transition from direct-to-indirect bandgap are similarly observed in Figure 5C. As for compressive strain (i.e., negative strain in Figure 5C), direct-to-indirect bandgap transition is observed at a strain of −1.5%, which is similar to previous reports with MoS_2_ (Chang et al., 2013; Yun et al., 2012; Muoi et al., 2019). The bandgap changes and its direct-to-indirect transition could be explained by strain-induced modification of the coupling strength between atomic orbitals in TMDs. Accordingly, we demonstrate the orbital nature of electronic band structures of WS_2_, such as d_z_2, d_xy_, and d_x_2-_y_2, in Figure 5. The direct-to-indirect bandgap transition with such decreased bandgaps explains our experimental findings in the Raman and PL spectroscopy: our core-shell-structured WS_2_ monolayer was under strain, resulting in the lateral modulation of the bandgap (Wang et al., 2015; Conley et al., 2013; Lu et al., 2012; Kumar and Ahluwalia, 2013).

### Conclusion

We investigated WS_2_ monolayers grown by CVD. Certain flakes exhibited a multi-domain structure, which was similar to the previously reported core-shell structure of MoS_2_. However, the control of the quantity of W allowed for a novel type of core-shell structure in the WS_2_. The core-shell growth mode in our CVD provided a lateral electronic heterostructure to modulate the bandgap of WS_2_ in a geometry with two domains: a core and an S-deficient shell. The two domains showed distinct Raman and PL spectra. The core-shell-structured WS_2_ monolayer exhibited PL spectra with a variation of optical bandgap at approximately 9.4% with a broad, weak, and blue-shifted PL peak. Our DFT calculations showed that the optical bandgap of the WS_2_ monolayer can be decreased by tensile strain on the core domain of the WS_2_ monolayer using a direct-to-indirect bandgap transition.

### Limitations of the study

In our study, the core and shell domains in WS_2_ monolayers have distinct bandgaps, resulting in optically distinguishable images of the core and shell domains in WS_2_ monolayers. Therefore, we observed core-shell structures in WS_2_ by optical microscope, and around 4 out of 10 flakes have such clear core-shell structures. We have tried to synthesize WS_2_ monolayers uniformly with the core-shell structures. But the subtle CVD conditions make it hard to uniformly control the growth mode that requires locally insufficient supply of W and S sources. Therefore, further study to control the growth mode is required for homogeneous WS_2_ flakes with core-shell structures.

## STAR★Methods

### Key resources table

**Table:** 

REAGENT or RESOURCE	SOURCE	IDENTIFIER
**Chemicals, Peptides, and Recombinant Proteins**		

Ammonium metatungstate hydrate	Sigma-Aldrich	CAS: 12333-11-8
Sodium cholate hydrate	Sigma-Aldrich	CAS: 206986-87-0
OptiPrep™ Density Gradient Medium	Sigma-Aldrich	CAS: 92339-11-2
Sulfur powder	Sigma-Aldrich	CAS: 7704-34-9

**Other**		

Chemical Vapor Deposition	NanoTech Planet	http://www.ntplanet.co.kr/
Raman spectroscopy	Nanobase	https://www.nanobase.co.kr/
AFM	Hitachi	https://www.hitachi-hightech.com/global/science/products/microscopes/afm/

**Software and algorithms**		

OriginPro 9.0	OriginLab Corporation	https://www.originlab.com/
Vienna ab-initio Simulation Package (VASP)	Kresse and Furthmüller (1996)	https://www.vasp.at/

### Resource availability

#### Lead contact

Further information and requests for resource and reagents should be directed to and will be fulfilled by Lead Contact, Heejun Yang (h.yang@kaist.ac.kr).

#### Materials availability

All unique/stable reagents generated in this study are available from the Lead Contact with a completed Materials Transfer Agreement.

### Experimental model and subject details

Our study does not use experimental models typical in the life sciences.

### Method details

#### Sample synthesis

WS_2_ monolayers were grown by CVD on a SiO_2_ (300 nm)/Si substrate. Ammonium metatungstate hydrate ((NH_4_)_6_H_2_W_12_O_40_·*x*H_2_O (Sigma-Aldrich, ≥ 66.5% (W)) was used as a W source and sodium cholate hydrate (Sigma-Aldrich) was used as a promoter, which were dissolved in DI water separately. Then, they were well-mixed with a medium solution (OptiPrep). After dropping the mixed solution onto the Si/SiO_2_ substrate, we conducted spin-coating process to spread the solution uniformly (Jeong et al., 2017; Yun et al., 2015). S powder (Sigma Aldrich, ≥ 99.95 %) was supplied continuously during the CVD process. A two-zone furnace was separately heated with a steady flow of Ar gas, 500 standard cubic centimeters per minute (sccm), to the set temperatures with a ramping rate: 200°C at 33°C/min for the S source and 770°C with 128°C/min for the SiO_2_/Si substrate with W. The temperature was maintained at the set temperature for 12 min, and afterward, the furnace was gradually cooled to room temperature.

#### Sample characterization

Atomic force microscopy (AFM) measurements were performed using a Hitachi AFM (5100N, Japan) in non-contact mode. Confocal Raman spectroscopy and PL spectroscopy were performed (XperRAM S series, Nanobase) with a wavelength of 532 nm for laser excitation. To avoid sample damage, we used an excitation laser power of less than 3 mW. All Raman peaks were calibrated using the Raman peak of Si located at 520 cm^−1^.

#### Computational method

The geometry of the WS_2_ structures with different strains was optimized using DFT as implemented in the Vienna ab-initio Simulation Package (VASP) (Kresse and Furthmüller, 1996) with projector augmented wave (PAW) pseudopotentials. All calculations were performed using the generalized gradient approximation (GGA) in the form of Perdew–Burke–Ernzerhof (PBE) (Perdew et al., 1996). We employed a cut-off energy for plane waves at 400 eV for all the calculations. All the atom positions were optimized until the convergence tolerance of the force was less than 0.001 eV/Å. A vacuum space of 10 Å in the z-direction was introduced to avoid interactions between adjacent periodic systems. The (24 × 24 × 1) Γ-centered Monkhorst–Pack meshes in the Brillouin zone (Monkhorst and Pack, 1976) were employed for optimization. Band structure calculations were performed along the high-symmetry path of M–K–Г–M. The equilibrium lattice parameters of *a* = 0.318 nm, and *c* = 1.311 nm were used to simulate the hexagonal (space group: P6_3_/mmc) WS_2_ monolayer. For the calculation of electronic structures modified by compression or tensile strain, we changed the lattice constants (*a* and *b*) with a portion from −2.0 to 2.0%, keeping the same volume with the small lattice constant changes.

### Quantification and statistical analysis

Our study does not include statistical analysis or quantification.

## Data Availability

Any additional information required to reanalyze the data reported in this paper is available from the lead contact upon request. No new code was generated during the course of this study.
