# Supplementary material for: Bandgap modulation in the two-dimensional core-shell-structured monolayers of WS2
Source: iScience. 2021 Dec 3;25(1):103563. doi: 10.1016/j.isci.2021.103563 (PMC8693456; doi:10.1016/j.isci.2021.103563)
Supplement: Document S1. Figures S1–S5 and Tables S1 and S2 [file mmc1.pdf]

iScience, Volume *25*

Supplemental information

Bandgap modulation in the two-dimensional core-shell-structured monolayers of WS_2

Seohui Kang, Yonas Assefa Eshete, Sujin Lee, Dongyeun Won, Saemi Im, Sangheon Lee, Suyeon Cho, and Heejun Yang

Supplemental Information

Supplemental Tables

			Position (cm^{-1})	Amplitude	FWHM (cm^{-1})
**Single-domain WS_2**		2LA	350.3	46366	7.4
		E'_{2g}	355.0	20063	9.3
		A_{1g}	417.6	9264	9.0
**Multi-domain WS_2**	*a*	2LA	347.6	82491	8.0
		E'_{2g}	355.0	30513	9.8
		A_{1g}	416.6	21767	8.7
	b	2LA	348.5	50443	8.5
		E'_{2g}	355.3	22133	11.8
		A_{1g}	416.3	34849	12.3
	c	2LA	348.4	61661	8.7
		E'_{2g}	354.1	30804	12.1
		A_{1g}	416.3	31208	8.1
	d	2LA	348.3	51054	8.7
		E'_{2g}	353.7	22330	10.5
		A_{1g}	416.4	20729	7.1

Table S1. Gaussian curve fitting of Raman peaks (2LA, E'_{2g}, A_{1g} and Si) represented in Figure 1C for the single-domain WS_2 monolayer and in Figure 3B for the positions marked in the inset of Figure 3B. The experimental data was fitted by least square fitting and their error ranges of peak position (and FWHM) was roughly deduced as $\pm 0.05\ cm^{-1}$.

			Position (nm)	Photon energy (eV)	Amplitude	FWHM (nm)
Single-domain WS_2		A	615.0	2.02	656912	18.4
		A^-	630.3	1.97	415636	31.5
		D	660.5	1.87	327020	65.9
Multi-domain WS_2	*a*	A	625.3	1.98	4355	33.8
		A^-	-	-	-	-
	b	A	631.7	1.96	21500	20.8
		A^-	644.0	1.92	14753	29.8
	c	A	675.9	1.83	4213	23.4
		A^-	685.1	1.81	3691	35.7
	d	A	677.2	1.83	2261	30.6
		A^-	-	-	-	-

Table S2. Fitting results of PL spectra in Figure 1D for the single-domain WS_2 monolayer and in Figure 4C for the multi-domains WS_2 monolayers. The experimental data was fitted by least square fitting and their error ranges of peak position (and FWHM) and photon energy were roughly deduced as $\pm$0.05 cm^{-1} and $\pm$0.005 eV, respectively.

Supplemental Figures

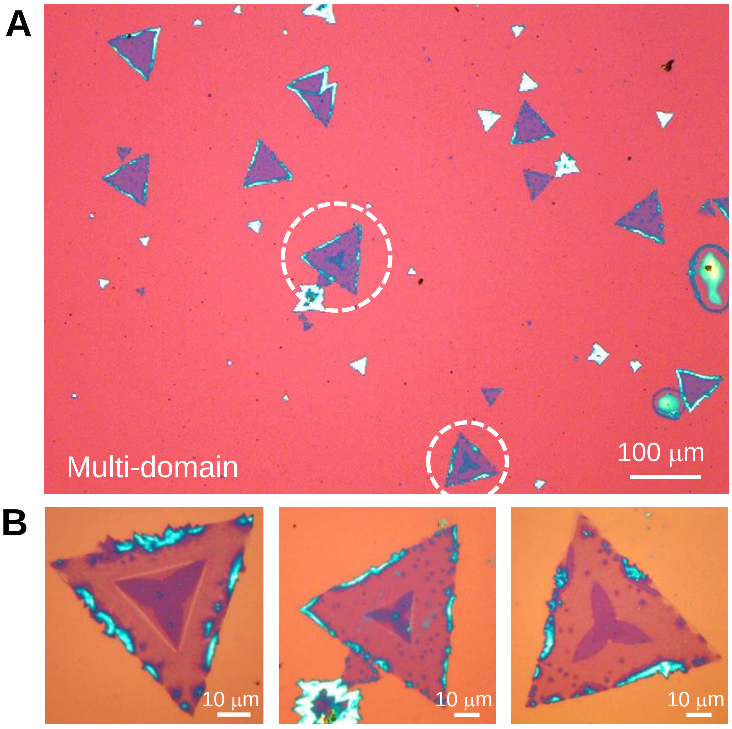

Figure S1. CVD grown WS_2 flakes on Si/SiO_2 substrate, related to Figure 2.

(A) Optical images of CVD grown WS_2 flakes on Si/SiO_2 substrate. The monolayer WS_2 flakes with multiple domains were marked with white dashed circles. **(B)** Optical images of multi-domain WS_2 monolayer, which were found in the same substrate.

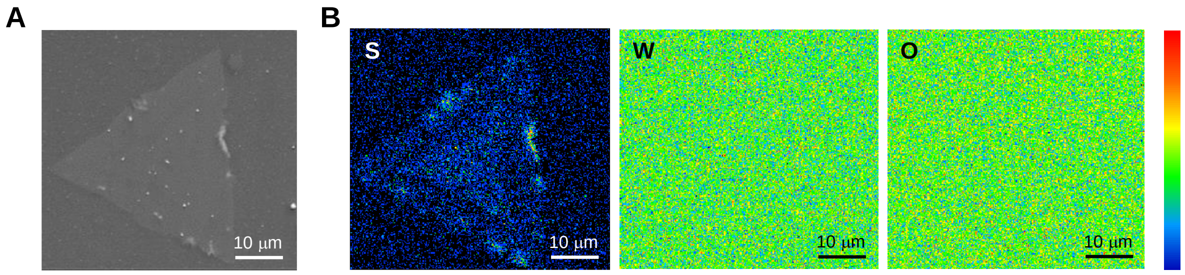

Figure S2. EPMA analysis of core-shell WS_2 monolayer, related to Figure 2.

(**A**) SEM image. (**B**) EPMA mapping images for S, W and O.

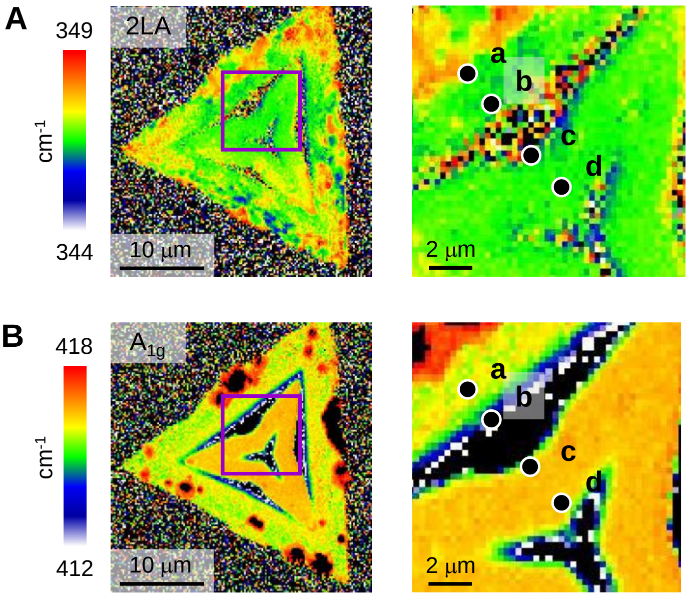

Figure S3. Raman shift mapping images and their enlarged images marked with locations of *a*, *b*, *c*, and *d* in the inset of Figure 3B.

(**A**) Mapping image with Raman modes of 2LA and (**B**) A_{1g} peak.

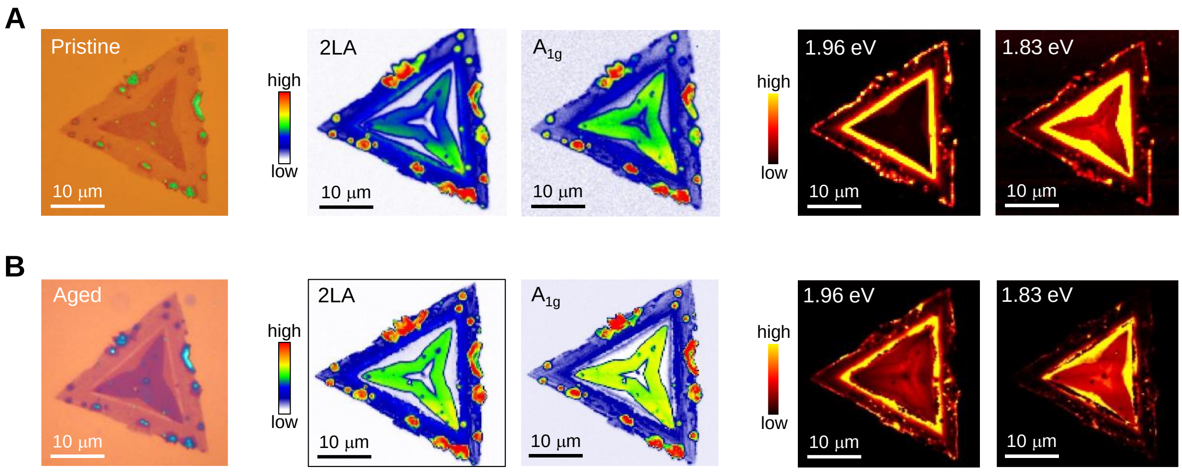

Figure S4. Comparison as-grown multi-domain WS_2 with aged multi-domain WS_2.

Optical image, Raman mapping with the peak position of 2LA and A_{1g}, and PL intensity mapping with two photon energies, 1.96 eV and 1.83 eV of (**A**) as-grown multi-domain WS_2 (August 2019) and (**B**) aged multi-domain WS_2 (October 2021) (related to **Figure 2**).

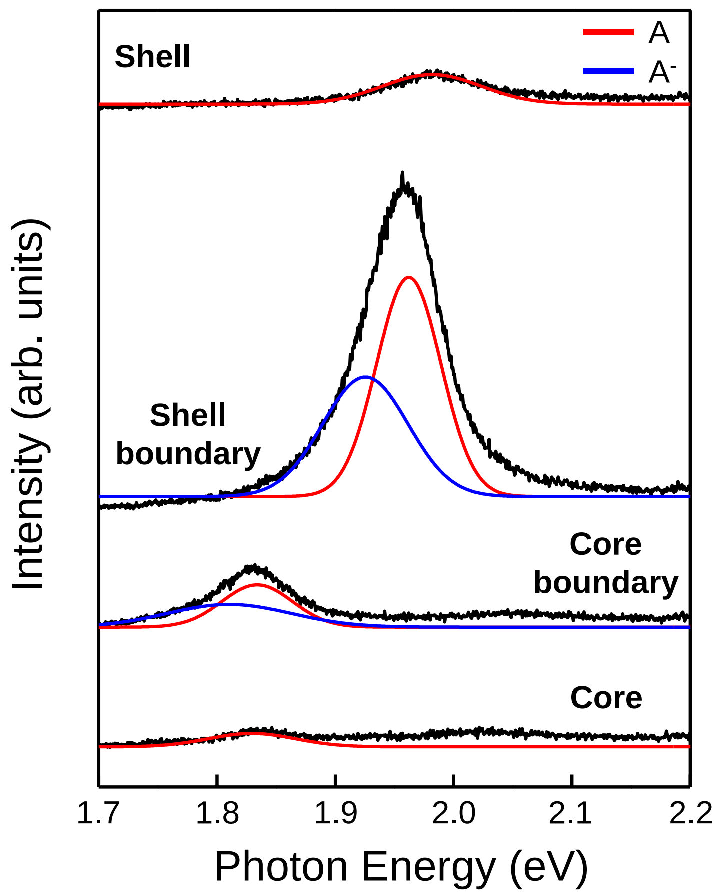

Figure S5. PL spectra with Gaussian fitting curves for real scale. The position *a*, *b*, *c*, and *d* are presented in the inset image of Figure 3B.
